# Orthorexia and perceived stress by medical students: which association?

**DOI:** 10.1192/j.eurpsy.2023.915

**Published:** 2023-07-19

**Authors:** R. Jbir, R. Masmoudi, M. Abdelkefi, F. Guermazi, I. Feki, R. Sellami, J. Masmoudi

**Affiliations:** 1 Psychiatry ‘A’, Hedi Chaker hospital university; 2 Hedi Chaker hospital universiry; 3 Hedi chaker hospital university; 4 Psychiatry ‘A’; 5 psychiatry A, Hedi chaker university hospital, Sfax, Tunisia

## Abstract

**Introduction:**

Orthorexia is a neuotic behavior characterized by an obsession with healthy eating. This trend is growing among medical students; it may be related to the stress experienced by these young people.

**Objectives:**

The aim of this study was to determine the prevalence of orthorexic eating behaviors among medical students in Tunisia and to examine the relationship with perceived stress.

**Methods:**

Our study was descriptive and analytical cross-sectional, carried out with medical students in the faculty of medicine of sfax (Tunisia) during October 2022.

An anonymous survey was asked to the students.

Data collection was done by a self-administered questionnaire via Google Forms administered in the students’ Facebook groups. The questionnaire was composed of a part for the collection of socio-demographic data and two psychometric scales :

-The ORTO-15 was used to assess orthorexia

- Cohen’s Perceived Stress Scale (PSS) to determine the level of stress

**Results:**

Atotal of 95 responses was collected.The average age of our samplewas 25.8±3.4 with sex ratio M/F=0,25.Tobacco and alcohol usewere found in 14.7% and 13.6% of casesrespectively.A psychiatric history was reported by 17.9% of students, 76.5% of whom are anxiety disorders.Average body mass index was 23.64 ± 3.53 kg/m2. More than half (58%)of the students were dissatisfied with their feed. In our sample, 8.4% of students have already consulted a nutritionist and 58.9% regularly practiced sport at gym.According to the ORTO 15, 52.6% of the students presented orthorexia. The mean score of the ORTO-15 was 39.19±4.48.According to PSS scores, 21.1% of students had severe level of stress, 69.5% had moderate stress level while 9.5% had low level of stress.Severe stress was significantly correlated with female gender and psychiatric follow (p=0.047), (p=0.001) respectively.Orthorexia was significantly correlated with the practice of sport (p=0.042).Orthorexics students had a higher level of stress without significant correlations.

**Conclusions:**

Our study showed significant frequencies of orthorexia and a considerable level of stress among medical students. A high level of stress was observed in these orthorexic students. Further studies should be conducted to better investigate this relationship in order to promote student mental health

**Disclosure of Interest:**

None Declared

